# Equine influenza virus at 70: What horses taught us about flu

**DOI:** 10.1371/journal.ppat.1014551

**Published:** 2026-08-28

**Authors:** Laura Mojsiejczuk, Thomas M. Chambers, Pablo R. Murcia

**Affiliations:** 1 MRC-University of Glasgow Centre for Virus Research, Institute of Infection, Immunity and Inflammation, College of Medical, Veterinary and Life Sciences, University of Glasgow, Glasgow, Scotland, United Kingdom; 2 Department of Veterinary Science, Maxwell H. Gluck Equine Research Center, University of Kentucky, Lexington, Kentucky, United States of America; Weill Cornell Medical College: Weill Cornell Medicine, UNITED STATES OF AMERICA

This year marks the 70th anniversary of the first documented isolation of equine influenza virus (EIV). In May 1956, major outbreaks of respiratory disease in horses took place in the former Republic of Czechoslovakia. The presence of antibodies against influenza A in serum from affected horses, together with observed similarities in clinical course with influenza in humans, led Sovinova and colleagues to “investigate the problem in greater detail”. Most nasal washes cultured in embryonated chicken eggs failed due to contamination, but sample 262 remained sterile, yielding A/equine/Prague/56 (H7N7) [1], marking the start of EIV research and initiating a new area of influenza research. While poultry and swine are also kept in dispersed, interconnected populations, horses uniquely combine those characteristics with a long lifespan and global mobility (trailing only humans as the most common commercial air travellers, for breeding and performance purposes), an intersection of epidemiological features that more closely resembles that of humans and makes EIV a particularly informative model system. This Pearl article is our personal view that aims to highlight how studies on EIV have informed fundamental concepts of influenza virus biology, evolution, and epidemiology (Fig 1).

**Fig 1:**
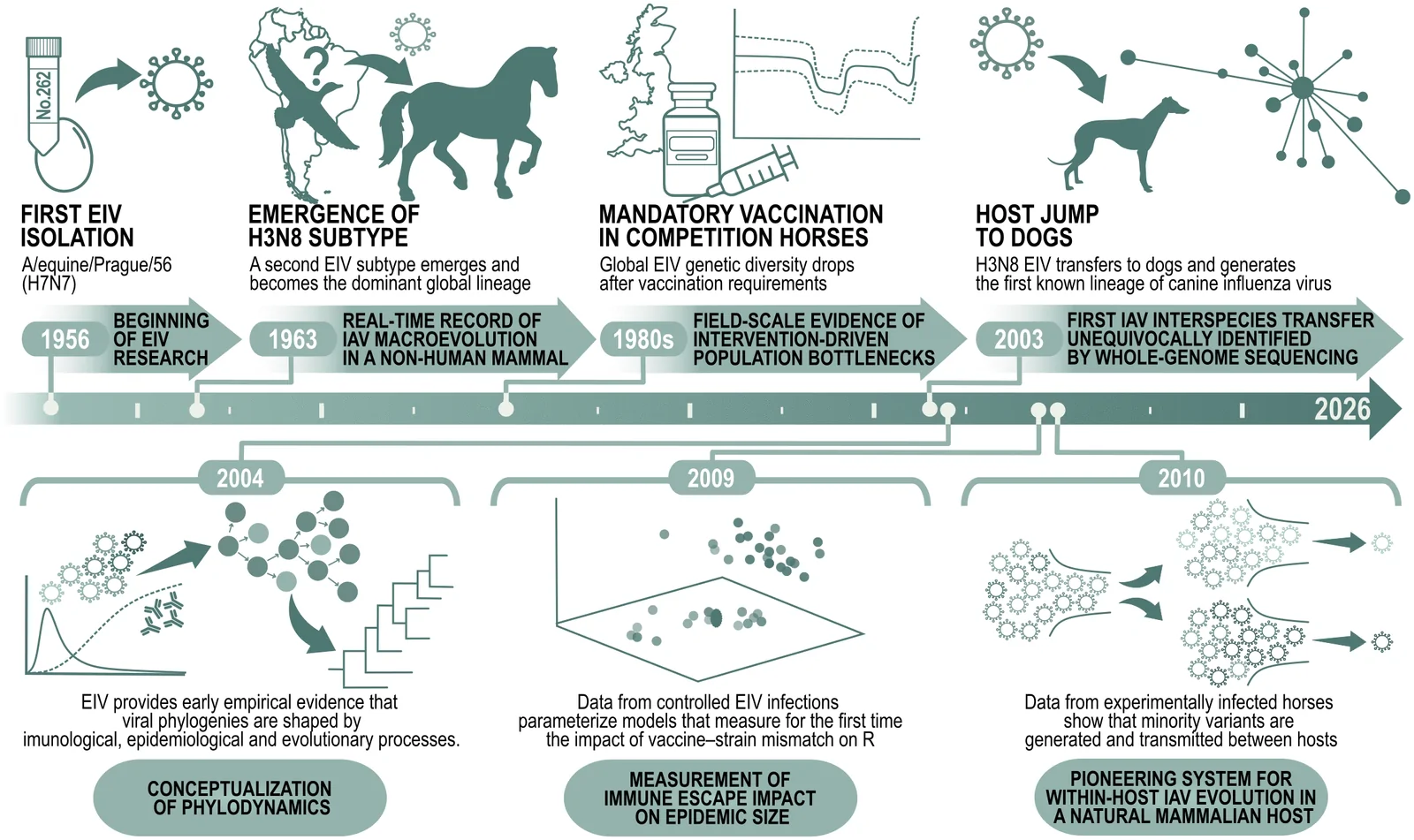
Seventy years of equine influenza virus research and its impact on virology. Timeline showing EIV-specific research milestones (upper track) and corresponding contributions to broader influenza and virology concepts (lower track) from 1956 to the present, which have shaped modern understanding of influenza across multiple host species. Icons adapted from Pixabay, PhyloPic.org, and Wikimedia Commons. Abbreviations: EIV, equine influenza virus; IAV, influenza A virus; R, effective reproduction number.

## Horses played a role in the history of human influenza

Influenza A viruses (IAVs) exhibit a complex ecology rooted in their natural avian reservoir, from which multiple cross-species transmission events have led to documented spillover and host shifts from birds to mammals and also among mammalian species. Major human pandemics have arisen from such processes. Within this context, horses have historically been considered as potential sources of zoonotic infections. Influenza has been recognised as a disease of the horse since 1299 AD. Before the invention and widespread use of the automobile, contact rates between people and horses were much higher because horses were the main means of transport. While empirical evidence is lacking, it has been suggested that horses played a role in the genesis of epidemic influenza [2] because human influenza epidemics were usually preceded by equine influenza outbreaks (importantly, this pattern disappeared as horses were replaced by cars). Indeed, a recently published hypothesis posed that horses may have contributed to the genesis of the virus that caused the 1918 pandemic [3], challenging conventional accounts.

A second EIV subtype -this time an H3N8 virus- emerged in 1963 [4]. In 1965, Minuse and colleagues reported serological evidence of cross-reactive anti-H3 antibodies in individuals born between 1874 and 1891, predating the emergence of H3 IAVs in humans and pigs [5]. These findings supported the idea, already under discussion at the time, that influenza virus subtypes may “recycle” and that animal reservoirs could play a role in this process.

## EIV revealed the hidden diversity within individual hosts

H3N8 EIV was used as a model system in pioneering studies aiming to elucidate the patterns of influenza within-host diversity and evolution. For decades, RNA viruses had been recognised as mixtures of genetic variants whose frequencies change rapidly during infection [6]. At a time when next-generation sequencing was in its infancy, clonal sequencing was combined with *in vivo* experiments in horses to measure for the first time the intra-host diversity of a mammalian IAV in its natural host. By identifying and counting mutations in the haemagglutinin gene of infected horses, those studies showed that acute equine influenza infections generate genetically distinct viral subpopulations beyond the consensus sequence and that most naturally arising mutations are deleterious [7]. It was also observed that some minority variants can persist for multiple days and even be transmitted, suggesting that virus transmission bottlenecks were not as narrow as previously perceived. These findings were later confirmed in natural outbreaks [8], also using clonal sequencing.

EIV intra-host data contributed empirical evidence for the development of a within-host evolutionary model that, applied to H5N1 in the ferret transmission model, showed that respiratory droplet-transmissible variants can arise during a single mammalian infection, yet remain below consensus detection thresholds, and that the proportion reached by these variants determines the likelihood of onward transmission [9]. Since those early EIV studies based on the analyses of only a few thousand haemagglutinin sequences, advances in sequencing technologies (currently reaching millions of sequencing reads covering the entire genome) and bioinformatic pipelines have refined this picture, confirming that most within-host variants are short-lived, with strong purifying selection and genetic drift dominating evolutionary dynamics during acute infection. Furthermore, empirical estimates in large human cohorts suggest relatively tight transmission bottlenecks and limited evidence for sustained positive selection of antigenic variants during acute infection [10]. Comparative analyses across viral models (e.g., dengue virus and cytomegalovirus) suggest that such constraints are not unique to influenza [6].

## EIV linked epidemic patterns to the long-term evolution of influenza A viruses

Phylodynamics is a conceptual framework formalised by Grenfell and colleagues that integrates immunodynamics, epidemiology and evolutionary biology to explain how within-host processes and population-level epidemic dynamics jointly shape viral phylogenies [11]. This theoretical framework has massively increased our understanding of infectious disease dynamics. As noted by Bryan Grenfell during the Commemorative lecture of the 2022 Kyoto Prize in Basic Science, the study of epidemics of influenza in horses stimulated ideas that became central to the theories in phylodynamics [12]. Data from experimental infections in horses were used to parameterise viral shedding and immune responses, enabling quantification of the impact of viral fitness in the context of host immune status and determination of transmission probability [11].

EIV has also informed our understanding of long-term IAV evolution. Worobey et al. reconstructed the evolutionary history of IAV across multiple hosts and identified a synchronised global sweep of the internal genes of avian influenza viruses in the nineteenth century [13]. Notably, their analyses placed equine H7N7 as a basal sister lineage to avian, human, swine, and equine H3N8 clades. Interestingly, the branch that separates those sister lineages covers the 1866–1878 period, which includes the major equine influenza epizootic of 1872 and leads Worobey and colleagues to speculate that “some epidemiological event” associated with that epizootic might have precipitated the global sweep of internal IAV genes. However, this proposal remains controversial: the back-extrapolation of any causal role for EIV or H7N7 in the broader emergence of mammalian and human influenza lacks direct empirical support.

## EIV documented decades of viral evolution in real time

EIV offers a remarkably complete, real-time record of influenza virus macroevolution in a nonhuman mammal, from a putative avian spillover in 1963 that led to the emergence of H3N8 EIV (and subsequent replacement of the H7N7 subtype), through decades of enzootic diversification and competitive lineage turnover, and including a second host jump into dogs [14]. Canine influenza virus (CIV) was first reported in racing greyhounds in 2004. Unusually among mammalian influenza viruses, it emerged through the direct mammal-to-mammal transfer of an entire nonreassortant equine virus that then adapted to and circulated enzootically in dogs; it remains the first documented interspecies transfer with the donor species unequivocally identified by whole-genome sequencing. Sustained mainly by dense, interconnected shelter and kennel dog populations, H3N8 CIV never spread beyond North America, was last detected in 2016, and is presumed to be extinct [15].

Phylogenetic analyses of global H3N8 EIV complete genomes showed that distinct clades can co-circulate for extended periods, and that intra-subtype reassortment is relatively frequent, in addition to typical diversification driven by gradual substitutions on a single lineage observed in human IAVs. Notably, H3N8 EIV evolved at a slower rate than other mammalian IAVs, potentially due to a weaker immune selection in its host population [16].

## EIV taught us practical lessons about influenza control and eradication

EIV research has also contributed to our general understanding of influenza epidemiology. Vaccine studies by the late Jennifer Mumford at the Animal Health Trust in Newmarket, United Kingdom, provided important information regarding vaccine composition (in terms of viral strains to be included as well as adjuvants) and duration of vaccine-mediated immunity, which in turn informed vaccination schedules. While this research contributed significantly to the development of measures to control equine influenza, data generated by those highly controlled experimental infections informed epidemiological models that explicitly measured, for the first time, how amino acid differences between immunising and circulating influenza strains impact on the effective reproductive number R, and as a result on epidemic size [17].

Beyond controlled experiments, population-level genomic analyses revealed that EIV displayed a significant drop in global genetic diversity after vaccination became mandatory in competition horses in the early 1980s, providing a field-scale example of interventions causing measurable population bottlenecks [16]. In Australia, a large EIV outbreak in 2007 affected more than 70,000 horses, but the virus was later eradicated by a combination of zoning, movement restrictions, vaccination (using DIVA vaccines, see below) and increased biosecurity [18]. To the best of our knowledge, this remains the only documented case of active eradication of a mammalian IAV following widespread introduction into a country. A recent study suggests that EIV could be eradicated from the United Kingdom, as continuous endemic transmission might not be supported [19].

As with human IAVs, vaccination is the best prophylactic measure recommended- and sometimes required- for horses at risk of exposure, such as by travel to competitions. Several EIV vaccine platforms are available, including inactivated virus, ISCOM-based, modified-live virus, and recombinant canarypox-vectored vaccines, and plant-based virus-like particles expressing HA, which offer a potentially viable alternative, as they are easier to update and produce [20]. The recombinant canarypox-vectored vaccines enable differentiation of infected from vaccinated animals (DIVA), a capability that was essential during the 2007 epizootic eradication campaign in Australia. EIV undergoes antigenic drift, though at a slower rate than human IAVs. Furthermore, vaccine strains are updated less frequently than those for human seasonal influenza, likely due to limited virological and economic incentives associated with the relatively small veterinary market.

## Conclusion

In sum, the study of EIV has made a significant contribution to our current understanding of influenza virus infections not only in horses but also in other mammalian hosts (including humans), illustrating the importance of comparative approaches in virology research.
